# Probing the Role of Melanocortin Type 1 Receptor Agonists in Diverse Immunological Diseases

**DOI:** 10.3389/fphar.2018.01535

**Published:** 2019-01-14

**Authors:** Carl Spana, Andrew W. Taylor, David G. Yee, Marie Makhlina, Wei Yang, John Dodd

**Affiliations:** ^1^Palatin Technologies, Inc., Cranbury, NJ, United States; ^2^Department of Ophthalmology, Boston University School of Medicine, Boston, MA, United States

**Keywords:** melanocortin, melanocortin 1 receptor, alpha-melanocyte stimulating hormone, PL-8177, PL-8331, inflammatory bowel disease, experimental autoimmune uveitis, dry eye disease

## Abstract

**Background:** The melanocortin α-melanocyte stimulating hormone (α-MSH), an endogenous peptide with high affinity for the melanocortin 1 receptor (MC1r), has demonstrated prevention and reversal of intestinal and ocular inflammation in animal models. Preclinical studies were performed to determine whether two MC1r receptor agonists, PL-8177 and PL-8331, exhibit actions and efficacy similar to α-MSH in preventing and reversing intestinal and ocular inflammation.

**Methods:** Both PL-8177 and PL-8331 were assessed in a Eurofins LeadProfilingScreen selectivity panel including 72 *in vitro* assays. PL-8177 and PL-8331 were evaluated in an *in vitro* assay using human whole blood stimulated by lipopolysaccharide to determine inhibition of tumor necrosis factor alpha (TNF-α); for comparison, adrenocorticotropic hormone (ACTH) and α-MSH were used as positive controls. PL-8177, dosed at 0.5, 1.5, and 5.0 μg, was assessed in a cannulated rat model of dinitrobenzene sulfonic acid (DNBS)-induced bowel inflammation versus vehicle and oral sulfasalazine. PL-8177 was also dosed at 0.3 mg/kg/mouse injected intraperitoneally versus untreated controls and α-MSH treatment in mice with experimental autoimmune uveitis (EAU). PL-8331 at 3 doses, 3 times daily, was evaluated in a murine model of scopolamine-induced dry eye disease (SiccaSystem^TM^ model), versus twice-daily Restasis^®^ and Xiidra^®^.

**Results:** Both PL-8177 and PL-8331 demonstrated no significant activity at the 1 μm concentration in any of the 72 *in vitro* assays. PL-8177 and PL-8331 inhibited lipopolysaccharide-induced TNF-α to a similar degree as ACTH and α-MSH. In the DNBS rat model of bowel inflammation, PL-8177 was significantly superior to untreated controls at all 3 doses (*P* < 0.05) in reducing bowel inflammation parameters, with effects similar to sulfasalazine. In the murine EAU model, PL-8177 significantly reduced retinal inflammation scores versus untreated controls (*P* = 0.0001) over 3–5 weeks, and to a similar degree as α-MSH. In the murine scopolamine-induced model of dry eye disease, PL-8331 reduced corneal fluorescein staining scores at all doses, significantly (*P* = 0.02) for the highest dose (1 × 10^-5^ mg⋅mL^-1^), and similarly to Restasis^®^; Xiidra^®^ demonstrated no effect.

**Conclusion:** The MC1r receptor agonists PL-8177 and PL-8331 exhibited actions similar to those of α-MSH in preventing and reversing intestinal and ocular inflammation in preclinical disease models.

## Introduction

The melanocortins are endogenous hormonal peptides that are cleaved from a precursor hormone called proopiomelanocortin (POMC), which was first found in the pituitary gland but is also expressed in the central nervous system and in diverse peripheral tissues (Catania et al., 2004; Brzoska et al., 2008). POMC neurons project to various hypothalamic areas and multiple other brain regions that regulate energy homeostasis, and are produced in cells of both the immune and neuroendocrine systems (Blalock, 1985, 1999; Catania et al., 2004). The melanocortins consist of 4 peptides: adrenocorticotropic hormone (ACTH), and α-, β-, and γ-melanocyte stimulating hormone (MSH) (Catania et al., 2004; Brzoska et al., 2008). While the adrenal stimulatory effects of ACTH and the role of MSH in pigmentation are well known, the melanocortins have also demonstrated pleiotropic *in vitro* and *in vivo* anti-inflammatory and immunomodulatory actions in a variety of tissues and organ systems (Catania et al., 2004, 2010; Brzoska et al., 2008).

A key development that allowed for greater knowledge of these diverse actions was cloning of the melanocortin receptor family, leading to the identification of 5 receptors (Mountjoy et al., 1992; Catania et al., 2010). ACTH selectively binds to melanocortin 2 receptor (MC2r). The other receptor subtypes [melanocortin 1, 3, 4, and 5 receptors (MC1r, MC3r, MC4r, and MC5r)] recognize all the other melanocortins, although with varying degrees of affinity (Brzoska et al., 2008; Catania et al., 2010). The receptors are expressed in a variety of cell types, such as monocytes, macrophages, neutrophils, and fibroblasts, and are distributed widely in the central nervous system and peripheral tissues, including the skin, gut, sex organs, lungs, heart, and liver, among other areas (Brzoska et al., 2008; Catania et al., 2010). The melanocortin receptors are involved in many physiological functions and protective actions against infection and disease, and appear to act primarily via the cyclic adenosine monophosphate (cAMP) pathway (Catania et al., 2010; Cai and Hruby, 2016). MC1r and MC3r have demonstrated anti-inflammatory effects; α-MSH and ACTH have the strongest affinity for MC1r, and γ-MSH and ACTH are the strongest ligands for MC3r (Suzuki et al., 1996; Catania et al., 2004; Brzoska et al., 2008).

Major anti-inflammatory mechanisms of α-MSH, demonstrated *in vitro* and *in vivo*, include inhibition of activation of the nuclear transcription factor NF-κB, which mediates expression of an array of pro-inflammatory cytokines, chemokines, and adhesion molecules (Manna and Aggarwal, 1998; Ichiyama et al., 1999; Hassoun et al., 2002; Catania et al., 2010), and upregulation of the cytokine synthesis inhibitor interleukin-10 (Luger et al., 2003; Raap et al., 2003). α-MSH has also been shown to promote the *in vitro* regulatory activity of T-cells and their conversion into Treg cells (Taylor et al., 2000; Taylor and Lee, 2011). In addition, *in vitro* studies suggest that α-MSH inhibits tumor necrosis factor-alpha (TNF-α) and nitric oxide production, as well as other anti-inflammatory effects, via an autocrine regulatory circuit in macrophages, which counteracts the effects of pro-inflammatory cytokines and contributes to the resolution phase of inflammation (Star et al., 1995; Rajora et al., 1996; Taherzadeh et al., 1999; Yoon et al., 2003; Li and Taylor, 2008). Resolution is characterized by cessation of the inflammatory response with phagocytosis of apoptotic neutrophils, sequestration of pro-inflammatory cytokines, clearing of pathogens and debris, healing and repair, and return to homeostasis (Patel et al., 2011; Serhan, 2017). Emerging evidence suggests that several melanocortins promote processes of resolution via receptors in macrophages (Patel et al., 2011).

Based on these data, researchers have investigated the anti-inflammatory and pro-resolution effects of novel melanocortin receptor agonists in treatment of chronic inflammatory or autoimmune conditions (Ahmed et al., 2013; Montero-Melendez et al., 2015; Cai and Hruby, 2016). Among these, PL-8177 and PL-8331 (Palatin Technologies, Inc.) are potent MC1r receptor agonists that demonstrate binding characteristics similar to those of α-MSH when evaluated against MC1r receptors (Spana et al., 2018; Table 1). Studies in preclinical models have shown that MC1r receptors in the colon played a pivotal role via the α-MSH pathway in the endogenous clearance of the inflammatory response to colitis (Maaser et al., 2006), and that α-MSH treatment reduced parameters of intestinal inflammation and promoted healing in experimental disease models (Kannengiesser et al., 2008; Yoon et al., 2008; Wei et al., 2016). Melanocortins also maintain immune homeostasis in the healthy eye (Caspi, 1999; Clemson et al., 2017), and preclinical studies found that α-MSH suppressed autoimmune inflammation associated with experimental uveitis (Taylor et al., 2000; Lee et al., 2009; Clemson et al., 2017). Therefore, PL-8177 and PL-8331 were evaluated in a series of preclinical studies to evaluate whether they exhibit actions similar to α-MSH in preventing and reversing intestinal and ocular inflammation.

**Table 1:** *In vitro* MC1r activity of endogenous melanocortins and Palatin (PL) MC1r agonists.

Activity	Alpha-MSH	ACTH	PL-8177	PL-8331
MC1r binding affinity (nM)	0.095	4.0^a^	0.04	0.01
MC1r functional activity (cAMP production, nM)	0.22	980	0.39	0.033


*ACTH, adrenocorticotropic hormone; cAMP, cyclic adenosine monophosphate; MC1r, melanocortin-1 receptor; MSH, melanocyte-stimulating hormone, ^a^Schioth et al. (1997).*

## Materials and Methods

### Agents Used in Studies

PL-8177 binds selectively to the MC1r while PL-8331 is a pan-agonist of melanocortin receptors. The binding characteristics of these agents for melanocortin receptors other than MC1r are compared in Table 2. PL-8331 was selected for experiments in models of dry eye disease since there is evidence that the MC5r plays a key anti-inflammatory and protective role in the retina (Taylor et al., 2006; Clemson et al., 2017). As stated above, the breadth of potential therapeutic actions and benefits of selective MC1r agonism were of particular interest in this study, which prompted the use of PL-8177 in the distinct colonic and ocular disease models.

**Table 2:** Comparison of PL-8177 (A) and PL-8331 (B) Binding and cAMP Functional Activity at Melanocortin Receptors 2–5.^a^

	EC50 (nM)			Emax @ 10 uM (%)		
	Ave	Std	n	Ave	Std	n
**A. PL-8177**						
MC2r	10000	0	1	15	0	1
MC3r	10000	0	1	45	0	1
MC4r	510	0	1	87	0	1
MC5r	9700	424	2	45	8	2
**B. PL-8331**						
MC2r	10000	0	1	18	0	1
MC3r	1	0	1	72	0	1
MC4r	0.77	0	1	80	0	1
MC5r	15	1	2	50	9	2


*^a^Testing conducted and data generated by CEREP, Inc., a subsidiary of Eurofins Panlabs, Inc. EC50, half maximal effect; Emax, maximal effect; MC, melanocortin; r, receptor.*

### PL-8177 and PL-8331: Selectivity/Specificity Profile *in vitro* Assays

Both PL-8177 and PL-8331 were evaluated in a Eurofins LeadProfilingScreen selectivity panel of 72 assays. Key screens included activity for cytochrome P450 enzymes 1A2, 2C19, 2C9, 2D6, and 3A4; potassium channel hERG; and 7 adrenergic receptor subtypes. Activity at 1 μm was the primary measure.

### PL-8177: Inflammatory Bowel Disease Experimental Model Studies

The *in vitro* activity of PL-8177 was compared to that of ACTH and α-MSH for inhibition of lipopolysaccharide-induced TNF-α in human whole blood (*n* = 3). The primary measure was the percent stimulated control of TNF-α, and the unit of measure was log_10_ M.

The potential effects of PL-8177 for inflammatory bowel disease (IBD) were evaluated in a proof-of-principle study using a dinitrobenzene sulfonic acid (DNBS)-induced model of bowel inflammation. In this study, DNBS was administered rectally as a solution in male, 200 g Wistar rats to induce inflammation of the bowel lumen. The rats were implanted with a catheter in the proximal part of the ascending colon, which exited out the nape of the neck for dosing access. In groups of 10, the cannulated rats were each dosed with PL-8177 at 0.5, 1.5, and 5.0 μg, and with vehicle (sterile water), via intracolonic injection at 24, 12, and 2 h before, and 6 h after, the DNBS challenge. This initial regimen was followed by twice-daily dosing for 5 consecutive days through Day 7 of the study. An active control group of non-cannulated rats was administered peroral sulfasalazine (5-aminosalicylic acid), a standard treatment for IBD (Hvas et al., 2018), and an untreated control group of non-cannulated rats was administered vehicle. Outcomes for this study were the changes in normalized colon weight and percent difference in inflammation score in PL-8177-treated rats versus sulfasalazine-treated active controls and untreated controls at study end (Day 8). Scoring for ulcers/inflammation was as follows (Wallace et al., 1989): 0 = No damage; 1 = Focal hyperemia, no ulcers; 2 = One site of ulceration/inflammation <1 centimeter (cm); 3 = Two sites of ulceration/inflammation <1 centimeter; 4 = Major site(s) of ulceration/inflammation >1 cm; 5+ = Damage >2 cm, score increased by 1 for each additional cm of damage.

All aspects of this work including housing, experimentation, and animal disposal were performed in general accordance with the “Guide for the Care and Use of Laboratory Animals: Eighth Edition” (National Academies Press, Washington, D.C., 2011) in a Eurofins AAALAC-accredited laboratory animal facility. In addition, the animal care and use protocol was reviewed and approved by the Institutional Animal Care and Use Committee (IACUC) at Eurofins Panlabs Taiwan, Ltd.

### PL-8177: Experimental Autoimmune Uveitis Studies

Experimental autoimmune uveitis (EAU) was induced in 15 C57BL/6 mice by injecting an antigen emulsion of complete Freund’s adjuvant (CFA), with 5 mg/mL of desiccated *M. tuberculosis*, and 2 mg/mL of interphotoreceptor retinoid-binding protein peptide amino acids 1–20. The course of EAU was evaluated every 3–4 days by fundus examination. PL-8177 at the dose of 0.3 mg/kg/mouse was injected intraperitoneally into EAU study treatment mice (*n* **=** 5) on the first day of clinically positive uveitis (uveitis inflammation score ≥2) and 48 h after first dose. EAU mice used as positive controls (*n* **=** 5) were injected with two doses of native α-MSH on the same schedule as the PL-8177 doses, and untreated EAU mice served as negative controls (*n* **=** 5). The outcomes for this study were the uveitis inflammation scores and confirmatory visual evaluation of retinal histology among the treatment groups. Uveitis inflammation scoring was conducted every 2 or 3 days by fundus examination and scored on a 0–5 scale. The fundus examination was performed on non-anesthetized mice, and their pupils were dilated with 1.0% Tropicamide ophthalmic solution before the examination. Scoring was as follows (Kitaichi et al., 2005): 0 = No inflammation; 1 = Eyes with only white focal lesions of vessels; 2 = Eyes with linear vessel lesions, over less than half of the retina; 3 = Eyes with linear vessel lesions, over more than half of the retina; 4 = Eyes with severe chorioretinal exudates or retinal hemorrhages in addition to the vasculitis; 5 = Eyes with a subretinal hemorrhage or a retinal detachment. Retinal histology was performed at end of study using conventional hematoxylin and eosin staining at Excalibur Pathology (Norman, OK, United States). All animal use in this research was approved by the Institutional Animal Care and Use Committee of Boston University.

### PL-8331: SiccaSystem^TM^ Model for Moderate Chronic Dry Eye Disease

A total of 70 mice were used in the study, which included multiple tests and treatment groups. Dry eye disease was induced in naïve, wild-type C57BL/6 mice using a combination of scopolamine, administered by subcutaneously implanted osmotic minipumps, and exposure to a controlled desiccating environment (15 L/min airflow, 5% humidity) in SiccaSystem^TM^ induction cages for 10 days. Extension time for development of dry eye was allowed if needed. On Day 12, corneal epithelial damage was assessed with fluorescein staining (one microliter of 0.05% liquid sodium fluorescein applied to the conjunctival sac) and captured by taking a photograph using a Leica DM IRBE long working-distance microscope (Leica Microsystems, Buffalo Grove, IL, United States). The severity of corneal surface inflammation was rated by blindly scoring fluorescein puncta and patches as follows: absent, 0; slightly punctate staining, 1; strong punctate staining but not diffuse, 2; small positive plaque areas, 3; and large area fluorescein plaque, 4 (Raap et al., 2003).

Based on the fluorescein staining results, the mice were randomized into 7 treatment groups such that each group had a median corneal surface inflammation score of 2. The 7 groups (*n* = 10 per group) were then administered treatment as follows: Group 1, untreated; Group 2, vehicle; Groups 3, 4, and 5: PL-8331 dosed at 1 × 10^-8^ mg⋅mL^-1^, 5 × 10^-7^ mg⋅mL^-1^, and 1 × 10^-5^ mg⋅mL^-1^, respectively; Group 6, Restasis^®^ (cyclosporine ophthalmic emulsion) treatment; Group 7, Xiidra^®^ (lifitegrast ophthalmic solution) treatment. The study drug (PL-8331) was administered 3 × daily (8 am, 1 pm, and 6 pm), by topical application (10 μL) into the conjunctival sac. The reference compounds, Restasis^®^ (cyclosporine ophthalmic emulsion) and Xiidra^®^ (lifitegrast ophthalmic solution) were administered twice daily (8 am and 6 pm), also by topical application into the conjunctival sac.

Outcomes data were presented as a mean ± standard error of mean (SEM) or median ± interquartile range. Data were analyzed using the Kruskal-Wallis analysis of variance (ANOVA; comparison of more than 2 groups) for non-parametric data sets, or one-way ANOVA for parametric data sets. Differences were considered statistically significant when *P* < 0.05. All animals were treated in accordance with the Association for Research in Vision and Ophthalmology (ARVO) Statement for the Use of Animals in Ophthalmic and Vision Research and the EC Directive 86/609/EEC for animal experiments, using protocols approved and monitored by the Animal Experiment Board of Finland (Experimentica Ltd. animal license number ESAVI-803-2017).

## Results

### PL-8177 and PL-8331: Selectivity/Specificity Profile *in vitro* Assays

Both PL-8177 and PL-8331 demonstrated no activity at 1 μm in any of 72 Eurofins lead profile *in vitro* assays. Of particular note, the screening showed no activity with either drug for cytochrome P450 enzymes 1A2, 2C19, 2C9, 2D6, and 3A4; potassium channel hERG; or any of 7 adrenergic receptor subtypes.

### PL-8177: *In vitro* LPS/TNF–α

**FIGURE 1:**
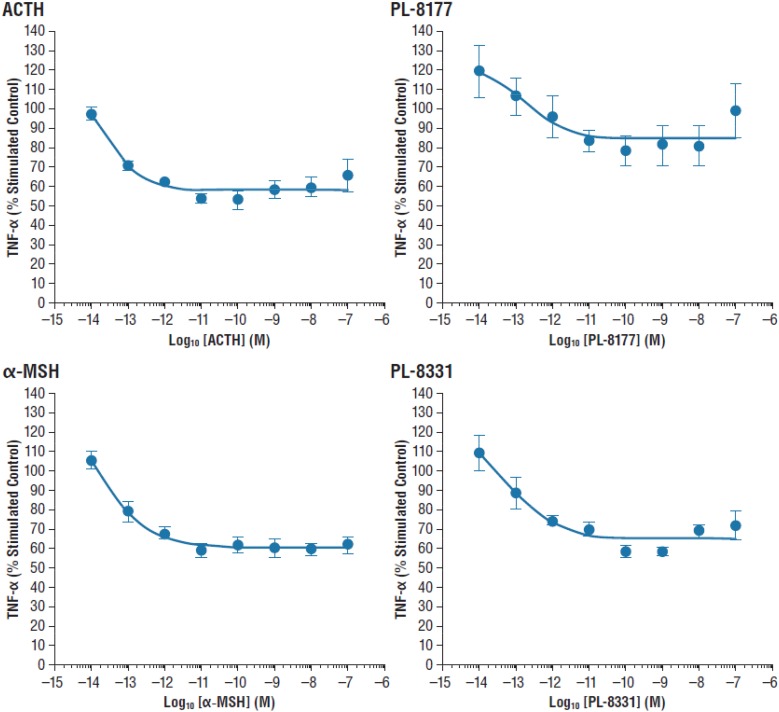
Inhibition of lipopolysaccharide-induced TNF-α inhibition in human whole blood. α-MSH, alpha-melanocortin stimulating hormone; ACTH, adrenocorticotropic hormone; TNF-α, tumor necrosis factor alpha.

In the *in vitro* study in human whole blood, MC1r selective agonist PL-8177 and PL-8331 inhibited lipopolysaccharide-induced TNF-α to a similar degree as the endogenous non-selective melanocortin receptor agonists ACTH and α-MSH (Figure 1).

### PL-8177: *In vivo* IBD Rat Model Study

**FIGURE 2:**
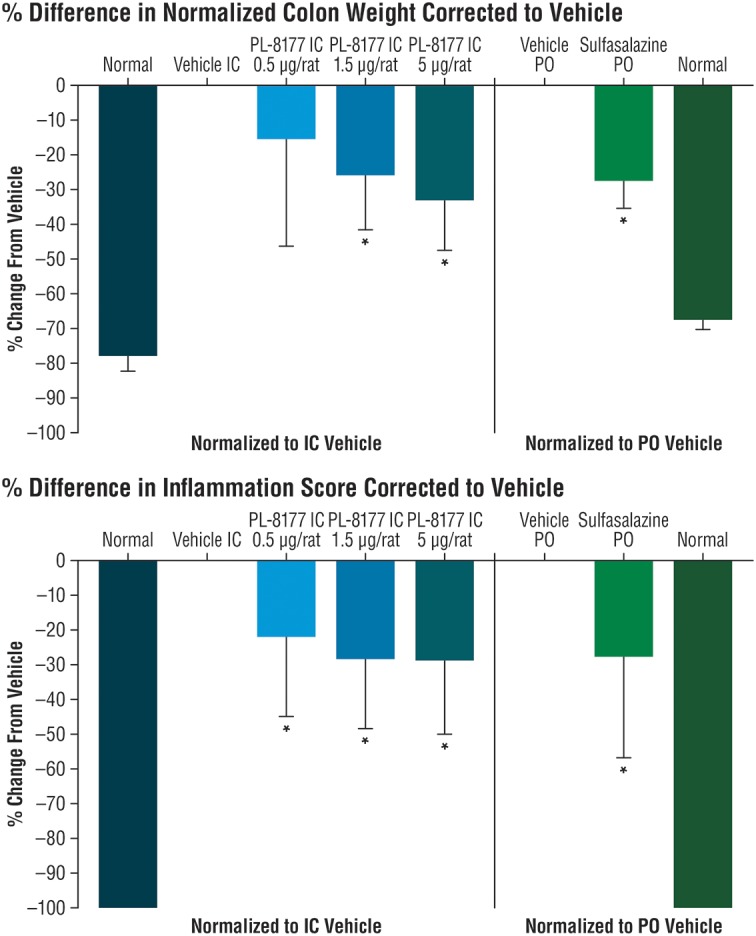
Effects of PL-8177 and sulfasalazine on colon weight and inflammation scores in rats with DNBS-induced bowel inflammation. ^∗^*P* < 0.05; DNBS, dinitrobenzene sulfonic acid; IC, intracolonic; PO, per oral.

In the proof-of-principle, cannulated rat model of IBD, PL-8177 was as active as the positive control sulfasalazine (standard IBD care) (Hvas et al., 2018), and was superior to untreated controls in reducing parameters of DNBS-induced bowel inflammation (Figure 2). A moderate dose-dependent effect was observed for the percent change in normalized colon weight corrected to vehicle; significant differences versus normal controls were shown for the PL-8177 1.5 μg/rat and 5.0 μg/rat doses (*P* < 0.05), but not for the 0.5 μg/rat dose (Figure 2). Significant differences versus normal controls (*P* < 0.05) were observed with all 3 PL-8177 doses for the percent difference in inflammation score corrected to vehicle, although effect sizes were slightly greater with the higher two doses (Figure 2).

### PL-8177: *In vivo* EAU Study

**FIGURE 3:**
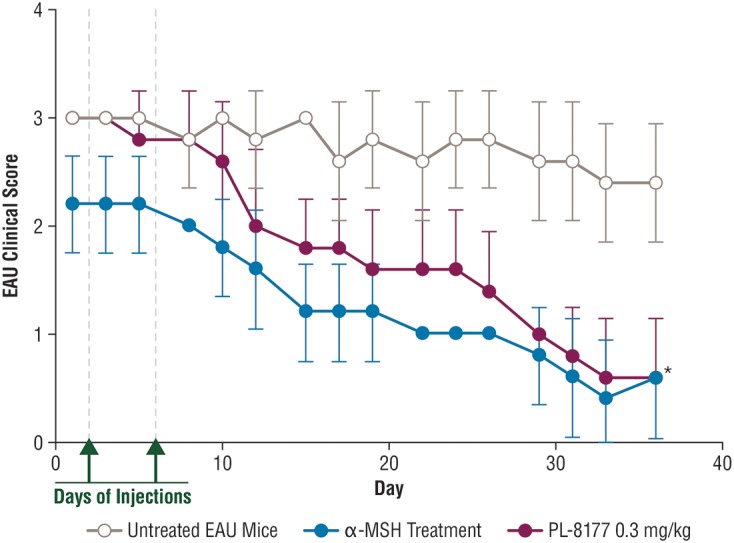
Effect of PL-8177 treatment versus controls on inflammation scores of mice with induced experimental uveitis. ^∗^*P* = 0.0001 by analysis of variance. α-MSH, alpha-melanocortin stimulating hormone; EAU, experimental autoimmune uveitis.

In the EAU study in C57BL/6 mice, 2 intraperitoneal injections of PL-8177 0.3 mg/kg/mouse given 48 h apart significantly reduced EAU inflammation scores versus untreated controls (*P* = 0.0001) over a 3- to 5-week period (Figure 3). The effects of PL-8177 in reducing EAU inflammation scores tracked closely with those of 2 α-MSH doses, also given 48 h apart, over the study period and merged with them toward the end, when scores in both active treatment groups were lowest (<1) (Figure 3). By contrast, the EAU scores in the untreated mice remained consistently near or at a score of approximately 3 throughout the study period.

**FIGURE 4:**
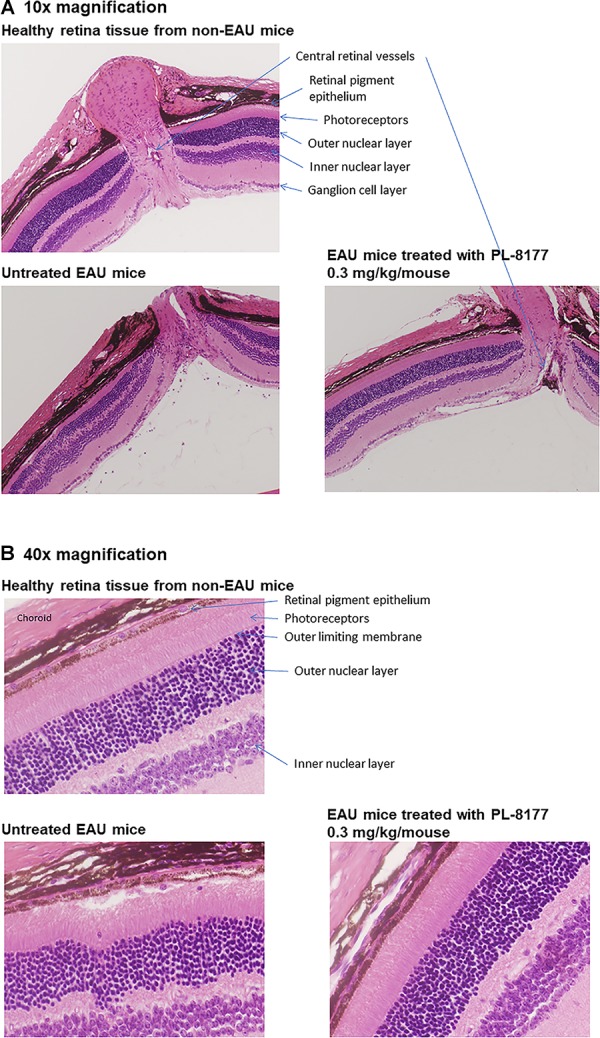
**(A)** Histology of retinas from healthy mice (Top), untreated EAU mice (Lower Left), and EAU mice treated with PL-8177 0.3 mg/kg/mouse (Lower Right). In contrast with the healthy retinas, the retinas from the untreated mice showed: cellular infiltration; uneven nuclear layers with folding; loss of the outer limiting membrane; loss of the intervening plexiform layer between the inner and outer nuclear layers in some places; a thinner photoreceptor layer, suggesting photoreceptor dropout; and signs of vasculitis in the central retinal vessels. **(B)** At higher magnification (40×), disruption of the retinal pigment epithelial monolayer and presence of immune cells in the photoreceptor layer were detectable. In contrast to the untreated retinas (Lower Left), EAU retinas treated with PL-8177 (Lower Right) retained the even layers of the retina with little evidence of photoreceptor loss [similar thickness of photoreceptor layer as in healthy eyes (Top)], and some of the outer limiting membrane. The retinal pigment epithelial monolayer was intact and there was a clear outer plexiform layer between the inner and outer nuclear layers. One detectable difference between the healthy retinas (Top) and the PL-8177-treated EAU retinas (Lower Right**)** is that photoreceptor nuclei were not lined up in the latter as they were in the healthy eyes.

Histology studies of the retinas of the mice in this study generally confirmed the EAU inflammation score findings (Figure 4). In comparison with healthy retinas from non-EAU mice, the retinas of untreated EAU mice showed cellular infiltration, uneven nuclear layers with folding, loss of the outer limiting membrane, and, in some places, loss of the intervening plexiform layer between the inner and outer nuclear layers. The photoreceptor layer was also thinner, suggesting photoreceptor dropout, and the central retinal vessels showed signs of vasculitis. At higher magnification, disruption of the retinal pigment epithelial monolayer and presence of immune cells in the photoreceptor layer were detectable. In contrast to the untreated EAU retinal tissue, EAU retinas treated with PL-8177 retained the even layers of the retina with little evidence of photoreceptor loss [similar thickness of photoreceptor layer as in healthy eyes], and some of the outer limiting membrane. The retinal pigment epithelial monolayer was intact and there was a clear outer plexiform layer between the inner and outer nuclear layers. One detectable difference between the healthy retinas and the PL-8177 EAU retinas is that photoreceptor nuclei were not lined up in the latter as they were in the healthy eyes.

### PL-8331: SiccaSystem^TM^ Model for Moderate Chronic Dry Eye Disease

**FIGURE 5:**
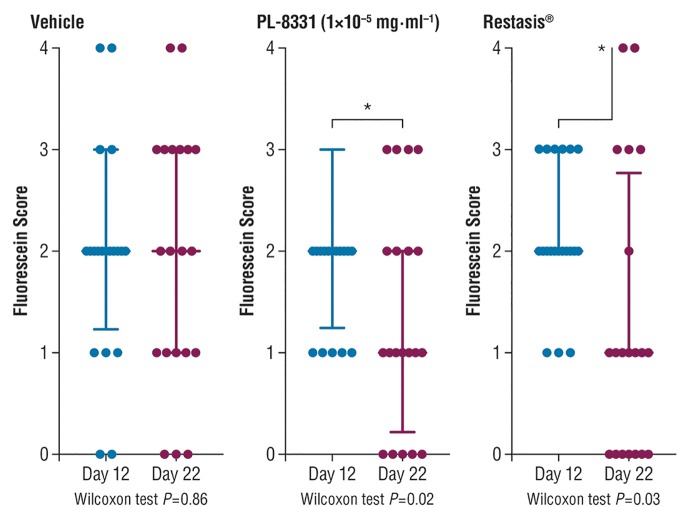
Corneal fluorescein staining in mice with chronic dry eye (SiccaSystem^TM^ model). Data are presented as median ± interquartile range; data werecompared by paired non-parametric Wilcoxon test. ^∗^Significant difference; see *P*-values under x-axis.

Corneal fluorescein staining scores for PL-8331 in a murine model of chronic dry eye disease showed reductions at all 3 doses (1 × 10^-8^ mg⋅mL^-1^, 5 × 10^-7^ mg⋅mL^-1^, and 1 × 10^-5^ mg⋅mL^-1^) on Day 22 versus Day 12 (Figure 5). The reduction was statistically significant (*P* = 0.02) on Day 22 versus Day 12 for the highest dose (1 × 10^-5^ mg⋅mL^-1^). This reduction was similar to that observed for Restasis^®^ (cyclosporine ophthalmic emulsion; reference treatment) on Day 22 versus Day 12 (*P* = 0.03). Xiidra^®^ (lifitegrast ophthalmic solution) had no effect on the treatment parameters studied.

**Table:** 

**Mean (SEM) values:**			

Day	Vehicle	PL-8331	Restasis
Day 12	1.95 (0.235)	2.05 (0.17)	2.15 (0.15)
Day 22	1.95 (0.285)	1.35 (0.244)	1.3 (0.309)


## Discussion

This series of preclinical studies confirmed that PL-8177 and PL-8331 exhibit the MC1r binding characteristics and pharmacologic actions of endogenous non-selective melanocortin receptor agonists in experimental models of immune-inflammatory conditions. Both PL-8177 and PL-8331 are potent MC1r receptor agonists that exhibited no significant activity at 1 μm in any of 72 *in vitro* assays in a Eurofins lead profile. The *in vitro* study in human whole blood showed that PL-8177 blocked lipopolysaccharide-induced TNF-α to a comparable degree as the endogenous melanocortin receptors ACTH and α-MSH. While blockade of TNF-α is an established anti-inflammatory mechanism of endogenous non-selective melanocortin receptor agonists, these hormonal peptides have multiple other mechanisms that may have equal or greater anti-inflammatory effects, as described in the introduction (Catania et al., 2004, 2010). Hence, this experiment established the similarity of PL-8177 to endogenous non-selective melanocortin receptor agonists in terms of a key anti-inflammatory mechanism, without indicating the relative overall effects of the agents compared to inflammation. For this reason, the studies reported here assess the anti-inflammatory effects of PL-8177 and PL-8331 by a variety of measures beyond that of TNF-α inhibition.

In a rat model of bowel inflammation, PL-8177 administered via catheter reduced inflammation and colon weight scores to a similar degree as sulfasalazine. Similarly, in a mouse model of autoimmune uveitis, PL-8177 administered via intraperitoneal injections significantly reduced retinal inflammation versus untreated controls, and to a similar degree as α-MSH. These therapeutic effects of PL-8177 were supported by histology studies of retinal tissue showing less damage from EAU in PL-8177-treated mice versus untreated controls. Although a difference in arrangement of photoreceptor nuclei was detected between the EAU retinas treated with PL-8177 and healthy retinas, this may have been a result of delayed treatment with PL-8177 following onset of uveitis. In addition, PL-8331 demonstrated reductions (significant at a dose of 1 × 10^-5^ mg⋅mL^-1^) in corneal epithelial damage due to dry eye, and similar to the effects of Restasis^®^, a comparator reference agent.

These findings contribute to a vast literature on the melanocortin systems and efforts to produce ligands for melanocortin systems that may provide useful drugs for an array of inflammatory, autoimmune, and degenerative conditions (Ahmed et al., 2013; Cai and Hruby, 2016). Given the broad distribution of melanocortin receptors in various tissues and organ systems, melanocortin receptor ligands, particularly α-MSH, and melanocortin receptor agonists have been studied in an array of preclinical models of inflammatory diseases including arthritis, liver inflammation, allergic airway inflammation, pancreatitis, brain inflammation, renal and lung injury, colitis, and ocular inflammation (Brzoska et al., 2008; Ahmed et al., 2013).

Among previous studies in experimental models of IBD, Rajora et al. (1997) found that administration of α-MSH in a mouse model of colitis reduced the appearance of fecal blood by over 80%, inhibited weight loss, and supported the general condition of the mice. The mice given α-MSH in this study also had markedly reduced production of TNF-α and nitric oxide in the lower colon. Oktar et al. (2000) reported that α-MSH reduced colonic lesions compared to untreated rats in models of both acute and chronic colitis. Maaser et al. (2006) further clarified the role of the MC1r receptor in experiments in mice with a frameshift mutation in the MC1r receptor gene (MC1r-Re/e mice) compared with C57BL/6 wild-type mice. In this study, the course of dextran sodium sulfate-induced (DSS) colitis was markedly more severe in the MC1r-Re/e mice, with significantly higher weight loss and worse histologic changes versus the C57BL/6 wild-type mice; the inflammation also led to death in all the MC1r-Re/e mice while all the C57BL/6 wild-type mice survived. In a subsequent study, Yoon et al. (2008) reported that α-MSH administered to BALB/c mice with DSS-induced colitis reduced symptoms of weight loss, colitis score, and histological damage and enhanced survival rate. Wei et al. (2016) conducted studies in a DSS rat model of ulcerative colitis using *Bifidobacterium* as a carrier to deliver α-MSH (*B. longum-α*-MSH) and thereby extend its activity, since the half-life of full-length α-MSH is only a few minutes *in vivo*. This study showed reduced activity of pro-inflammatory cytokines and histologic evidence of decreased inflammation and submucosal edema in the *B. longum-*α-MSH-treated group versus controls.

Variations of α-MSH treatment have also been studied in experimental models of uveitis and dry eye disease similar to those reported here. Lee et al. (2009) subconjunctively injected ACTH1-17, a naked plasmid that releases natively structured α-MSH peptide and no ACTH, in B10.RIII, C57BL/6, and MC5 knock-out mice with EAU induced by various methods. The ACTH1-17 plasmid treatment reduced the severity of EAU in the B10.RIII and C57BL/6 mice, but not in the MC5 knock-out mice, suggesting the anti-inflammatory action of this agent was dependent on MC5 expression. Ru et al. (2015) topically applied eye drops containing α-MSH at different doses to the corneas of rats with scopolamine-induced dry eye disease. This study found that the treatment increased tear secretion, enhanced tear film stability and corneal integrity, and reversed overexpression of pro-inflammatory factors including TNF-α, interleukin 1β, and type II interferon on the ocular surface. At the highest dose (10^-4^ μg/μL), the α-MSH drops exhibited protective effects on the corneal surface, suppressing apoptosis and restoring conjunctival goblet cells. Other studies have demonstrated protective, anti-inflammatory, and anti-apoptotic effects of α-MSH in the retina in rat models of diabetic retinopathy (Zhang et al., 2014; Cai et al., 2018). Potential protective mechanisms of α-MSH against diabetic retinopathy suggested by these studies include inhibition of *Forkhead box O* genes induced by high glucose concentrations (Zhang et al., 2014) and the correction of aberrant expression of inflammatory factors and tight junction genes, and inhibition of hyperpermeability, in diseased retinas (Cai et al., 2018). Investigation of whether PL-8177 and/or PL-8331 possess similar mechanisms and protective effects in preclinical models of diabetic retinopathy is of interest for future studies.

In the context of previous studies of α-MSH in experimental *in vivo* models of IBD and ocular inflammation, our present findings show that the MC1r receptor agonists PL-8177 and PL-8331 act in a similar manner to α-MSH in these disease models. Notable strengths of our studies include the addition of reference therapeutic drugs such as sulfasalazine, Restasis^®^, and Xiidra^®^ as comparators to the study drugs as well as α-MSH, in order to better characterize the effects and benefits of MC1r receptor-based therapies. In addition, this series of studies demonstrates the efficacy of MC1r receptor agonism across the different body systems evaluated. As peptides, the route of administration is important to be sure to achieve maximal efficacy. In two of the three models of inflammation discussed in this paper, local administration to the target tissue appears to be sufficient for obtaining appropriate levels of response.

Areas and questions of interest for future studies of PL-8177 and PL-8331 include more precise, molecular characterization of their effects with regard to the full spectrum of stages of inflammation and its resolution, and such studies are underway. Although the resolution phase of immune-inflammatory disorders is an area still under study, molecular resolution circuits and indices of resolution have been hypothesized, including maximal neutrophil numbers present during the inflammatory response; the time to occurrence of maximal neutrophil numbers; and the time to half maximal neutrophil numbers (Bannenberg et al., 2005). Evaluation of PL-8177 and PL-8331 with regard to these or other hypothesized parameters of resolution may help clarify the nature of their pharmacologic actions and potential clinical utility in prevention and treatment of immune-inflammatory disorders. Other challenges concerning MC1r agonists for clinical use include the risks of adverse events previously seen with such agents in clinical settings, including transient blood pressure increases and effects on skin, primarily hyperpigmentation (Ericson et al., 2017). Endogenous melanotropin receptors are also metabolically unstable and rapidly degraded, with half-lives of only several minutes *in vivo* (Ahmed et al., 2013; Cai and Hruby, 2016; Wei et al., 2016). Hence, MC1r mimetics must be designed to have enhanced bioavailability. PL-8177 and PL-8331 have longer terminal half-lives upon systemic administration than α-MSH (unpublished results) which allows greater opportunity for cell activation.

In summation, this series of studies demonstrated that the MC1r receptor agonists PL-8177 and PL-8331 demonstrated anti-inflammatory and protective actions similar to those of α-MSH and reference therapeutic drugs in animal models of intestinal and ocular immune-inflammatory disorders.

## Author Contributions

WY compiled the data used in the manuscript pertaining to *in vitro* information (Materials and Methods, etc.) and reviewed and edited the data reported for PL-8177 and PL-8331. MM compiled the *in vivo* information (Materials and Methods, etc.) contained in the manuscript and reviewed and edited the entire manuscript for technical accuracy. CS contributed to the organization of research results presented in the manuscript, authored portions of the introduction and discussion, and edited the entire manuscript. JD contributed to the compilation of data, organizational layout, authored portions of the introduction and discussion, and edited the entire manuscript. AT contributed to the writing, reviewing, and editing of the entire manuscript and providing the data and analysis of the EAU experiments presented in the manuscript. DY conducted and analyzed the EAU experiments presented in the manuscript.

## Conflict of Interest Statement

AT conducted the PL-8177 uveitis study supported by a sponsored research agreement with Palatin Technologies, Inc. CS, MM, WY, and JD are employees of Palatin Technologies, Inc. The remaining author declares that the research was conducted in the absence of any commercial or financial relationships that could be construed as a potential conflict of interest.
